# Improving Cancer Targeting: A Study on the Effect of Dual-Ligand Density on Targeting of Cells Having Differential Expression of Target Biomarkers

**DOI:** 10.3390/ijms241713048

**Published:** 2023-08-22

**Authors:** Nayer Sultana, Allan E. David

**Affiliations:** Department of Chemical Engineering, Auburn University, Auburn, AL 36849, USA; nzs0066@auburn.edu

**Keywords:** dual-ligand targeting, nanomedicine, drug delivery, colon cancer, biomarker expression

## Abstract

Silica nanoparticles with hyaluronic acid (HA) and folic acid (FA) were developed to study dual-ligand targeting of CD44 and folate receptors, respectively, in colon cancer. Characterization of particles with dynamic light scattering showed them to have hydrodynamic diameters of 147–271 nm with moderate polydispersity index (PDI) values. Surface modification of the particles was achieved by simultaneous reaction with HA and FA and results showed that ligand density on the surface increased with increasing concentrations in the reaction mixture. The nanoparticles showed minimal to no cytotoxicity with all formulations showing ≥ 90% cell viability at concentrations up to 100 µg/mL. Based on flow cytometry results, SW480 cell lines were positive for both receptors, the WI38 cell line was positive for CD44 receptor, and Caco2 was positive for the folate receptor. Cellular targeting studies demonstrated the potential of the targeted nanoparticles as promising candidates for delivery of therapeutic agents. The highest cellular targeting was achieved with particles synthesized using folate:surface amine (F:A) ratio of 9 for SW480 and Caco2 cells and at F:A = 0 for WI38 cells. The highest selectivity was achieved at F:A = 9 for both SW480:WI38 and SW480:Caco2 cells. Based on HA conjugation, the highest cellular targeting was achieved at H:A = 0.5–0.75 for SW480 cell, at H:A = 0.75 for WI38 cell and at H:A = 0.5 for Caco2 cells. The highest selectivity was achieved at H:A = 0 for both SW480:WI38 and SW480:Caco2 cells. These results demonstrated that the optimum ligand density on the nanoparticle for targeting is dependent on the levels of biomarker expression on the target cells. Ongoing studies will evaluate the therapeutic efficacy of these targeted nanoparticles using in vitro and in vivo cancer models.

## 1. Introduction

Nanoparticles have received significant attention as targeted drug carriers for cancer due to their large surface area-to-volume ratio, ability to encapsulate therapeutics, and easy surface functionalization [1,2]. Additionally, some unique properties of tumors have been exploited to enhance targeting of nanoparticles for therapeutic or diagnostic applications [3]. Furthermore, ligand conjugated nanoparticles have been shown to have a high affinity towards cancer cells with overexpressed biomarkers [2,4,5]. This specific targeting can increase the concentration of drugs at the cancer site, improving treatment efficacy and reducing non-specific toxicity [1,6]. Compared to single-ligand targeting, the use of dual- and four-ligands was shown to significantly improve targeting of cancer metastasis in a 4T1 mouse model of breast cancer [7].

In the ideal case, the targeted biomarker would be exclusively and homogenously expressed by only and all cancer cells. However, targeted biomarkers are often also expressed on normal cells and cancer cells themselves may show broad expression levels—leading to undesired and/or suboptimal targeting [8]. One approach to improve differential targeting of cancer cells, within the complex mixtures of cells found in the body, is to develop nanoparticles that target multiple biomarkers [9]. In addition to selection of the multiple targeting ligands with which to decorate the particle surface, the surface density of these ligands has also been shown to be important for cell targeting [10,11]. Other works have shown that ligand conformation and surface density are important factors in determining the receptor-binding affinity of the particles, which directly impacts targeting efficiency [12,13]. A too-high ligand density can promote surface adsorption of proteins, which can lead to nanoparticle clearance by the immune system [14]. Optimization of ligand density is, therefore, necessary to improve cancer cell targeting and limit non-specific interactions [15]. 

**Figure 1:**
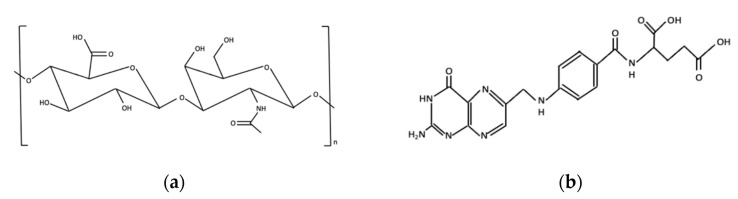
Chemical structure of (**a**) Hyaluronic Acid and (**b**) Folic Acid.

While other studies have compared dual- versus single-targeted nanoparticles, there is a significant gap in knowledge with regards to the effect of dual-ligand density and its optimization for targeting cells with variable biomarker expression. For example, Qhattal et al. presented that, compared to non-targeted control, particles with low ligand-grafting density of a single ligand showed no increase in targeting but the targeting increased significantly for particles with a high grafting density [16]. Another study by Moradi et al. showed increasing cellular targeting with higher surface ligand density up to a critical value above which it plateaued [17]. It has also been shown that dual ligands on the particle surface increase cellular targeting due to dual receptor-mediated endocytosis [18]. The goal of our study was to evaluate the impact of dual-ligand density on targeting of nanoparticles to cells with varying levels of biomarker expression. To achieve this goal, we developed silica nanoparticles conjugated with hyaluronic acid (HA) and folic acid (FA), shown in Figure 1, to target the Cluster of Differentiation 44 (CD44) and folate receptors, respectively, using a colon cancer model. As far as we are aware, this is the first study evaluating this dual targeted system for targeting of colon cancer cells. 

Silica nanoparticles were used as a model because of their biocompatibility, low toxicity, systemic stability, and relatively simple and low-cost preparation [19,20,21]. In addition, their controllable particle size, porosity, and crystallinity make them suitable for various biomedical applications [19]. Silica nanoparticles are ‘Generally Recognized As Safe’ (GRAS) by the United States Food and Drug Administration (US FDA) [22,23].

CD44, a single chain transmembrane glycoprotein [24,25,26], is overexpressed in many cancer cells, including ovarian, breast, lung, pancreatic, and colon [26,27], and it has a strong affinity towards hyaluronic acid (HA) [28]. Hyaluronic acid (HA), a natural linear polysaccharide made of alternating units of *N*-acetyl-d-glucosamine and D-glucuronic acid disaccharides [29,30], possesses excellent properties including biocompatibility, biodegradability, hydrophilicity, non-immunogenicity, and non-toxicity [26,31,32,33]. Folate receptor is a glycosylphosphatidylinositol (GPI) attached glycoprotein [34,35,36,37] that is also highly expressed in many cancers, including colon, breast, ovarian, kidney, lung, and brain. Folate receptors show high binding affinity (*K*_d_ = 1 nM) for folic acid and are an attractive target for drug delivery systems targeting cancer [38]. Folic acid (FA), also known as folate or vitamin B9 [39,40], possesses excellent properties including biocompatibility, high stability, non-immunogenicity, non-toxicity, simple conjugation chemistry, small size, and low cost [41,42]. 

In this work, we developed silica nanoparticles conjugated with HA and FA to target colon cancer. In vitro targeting studies were conducted using human colon cancer cells (SW480 and Caco2 cell lines) and lung normal cell (WI38 cell line), which were selected for their varying expression of CD44 and folate receptors. 

## 2. Results

### 2.1. Nanoparticle Synthesis and Characterization

**Figure 2:**
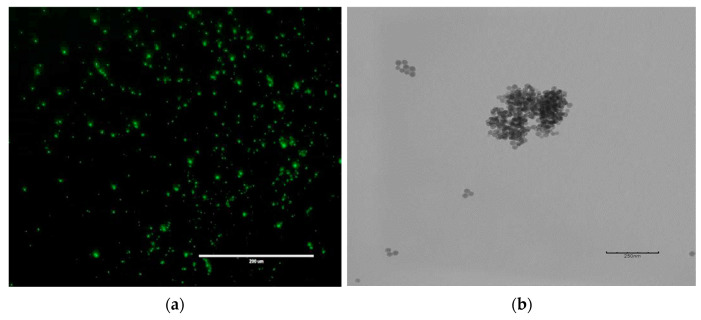
Typical nanoparticles produced in this study as visualized by (**a**) fluorescence microscopy and (**b**) TEM image. Particles showed bright fluorescence and uniform, spherical morphology.

Fluorescently labeled silica nanoparticles were synthesized using the water-in-oil microemulsion method, which is well-suited for formation of functional nanoparticles [43]. In our synthesis, the (3-aminopropyl)trimethoxysilane (APTMS):Tetramethyl orthosilicate (TMOS) mole fraction was fixed at 0.12 and the FITC concentration at 0.01M as higher amounts resulted in aggregation and fluorescence quenching, respectively. Figure 2 shows representative fluorescence microscopy and TEM images of the core silica nanoparticle. These particles showed excellent fluorescence and spherical morphology with relatively uniform size and served as the base for synthesis of the targeted nanoparticles.

Single- and dual-targeted nanoparticles were synthesized by modifying the surface of core nanoparticles with HA and FA. The ligand density on the particle surface was varied by adjusting the ligand-to-surface amine mole ratio during the chemical conjugation step. Four different hyaluronic acid-to-surface amine molar ratios (H:A), ranging from 0.5–1.25, and four folic acid-to-surface amine molar ratios (F:A), from 3–9, were selected for this study based on initial data (not shown) which demonstrated that they yielded nanoparticles with varying surface ligand densities. It was also observed that use of higher ratios led to nanoparticles that were prone to aggregate. Molar ratios of HA:EDC, HA:NHS, FA:EDC and FA:NHS were fixed at 1:50, 1:50, 1:2, and 1:2, respectively. 

Table 1 shows the average hydrodynamic diameter and polydispersity index (PDI) of targeted nanoparticles produced using the various H:A and F:A molar ratios. All data are reported as mean ± SD (*n* = 3). Nanoparticle sizes ranged from 150–250 nm with PDI in the range from 0.07–0.28. PDI provides an indication of the broadness of particle size distribution with values less than 0.1 generally considered to be monodisperse.

**Figure 3:**
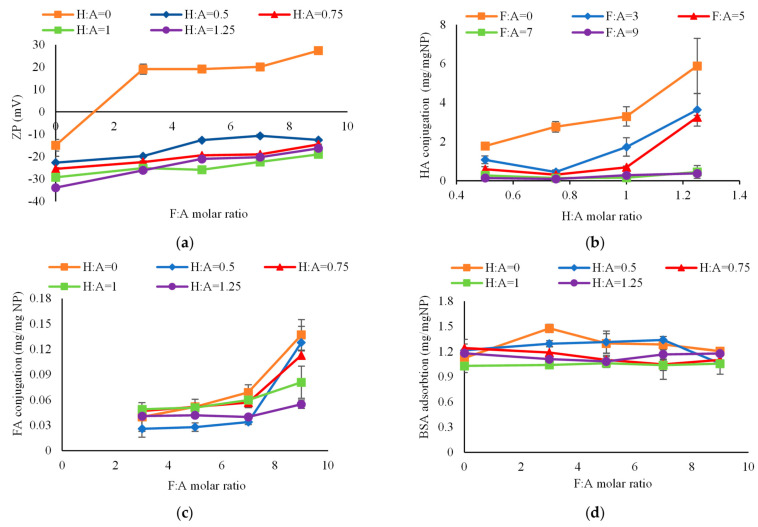
Physicochemical characteristics of targeted nanoparticles. (**a**) Effect of ligand conjugation on nanoparticle zeta potential. Zeta potential of the nanoparticles shifted toward positive direction with FA conjugation and reversed towards negative direction with HA conjugation; (**b**) Effect of H:A molar ratio on nanoparticle surface conjugation by HA. At lower F:A molar ratios, increase in H:A molar ratio significantly increased HA conjugation on nanoparticle surface; (**c**) Effect of F:A molar ratio on nanoparticle surface conjugation by FA. At lower H:A molar ratio, increase in F:A molar ratio significantly increased FA conjugation on nanoparticle surface; (**d**) BSA protein adsorption on nanoparticles. Ligand conjugation had negligible effect on protein adsorption.

Figure 3a shows the zeta potential of nanoparticles as a function of F:A molar ratio with each line representing a different H:A molar ratio. The average zeta potential of non-targeted, bare nanoparticles was −15 mV due to the presence of negatively charged silanol groups on the nanoparticle surface [44]. As expected, the zeta potential shifted towards the positive direction following modification with folic acid (orange line) due to the presence of protonated amino acid groups of folate [45]. Addition of hyaluronic acid produced a negative shift in zeta potential due to the presence of negatively charged, deprotonated carboxyl groups [46]. Zeta potential, therefore, provided some confirmation of the successful linkage of HA and FA to the nanoparticle surface. 

The amount of HA conjugated on the nanoparticles, as measured by the hexadecyltrimethylammonium bromide (CTAB) turbidimetric method [16], is shown in Figure 3b. CTAB is an anionic surfactant that forms an insoluble complex with polyanionic hyaluronic acid, which shows light absorption proportional to the HA concentration. It was observed that at higher F:A molar ratios (i.e., ≥7), there was no significant change in surface coverage by HA (*p* = 0.46 and 0.18 for FA:A = 7 and 9, respectively) as H:A was increased. At lower F:A molar ratios (i.e., ≤5), however, increase in:A molar ratio from 0.5 to 1.25 significantly increased HA conjugation on the nanoparticle surface (*p* = 0.037, 0.028, and 0.00001 for FA = 0, 3, and 5, respectively). With increasing F:A molar ratio, less HA was conjugated on the nanoparticle due to ineffective competition for reaction sites on the surface. Figure 3c shows the amount of FA conjugated on the nanoparticles as measured by spectrophotometry. As seen from the figure, at higher H:A molar ratios (i.e., ≥0.75), an increase in F:A molar ratio from 3 to 7 did not significantly change the FA surface coverage (*p* = 0.20, 0.36, and 0.13 for: A = 0.75, 1, and 1.25, respectively). However, further increase in F:A molar ratio to 9 significantly increased the surface coverage (*p* = 0.0009, 0.027, and 0.034 for H:A = 0.75, 1, and 1.25, respectively). At lower H:A molar ratios (i.e., ≤0.5), increase in F:A molar ratio from 0 to 9 significantly increased FA conjugation on the nanoparticle surface (*p* < 0.0001 and 0.0095 for H:A = 0 and 0.5, respectively). At F:A = 9, less FA was conjugated with increasing H:A molar ratio from 0 to 1.25, which again was likely due to competition for the reaction sites. 

Protein–nanoparticle interactions are important because the resulting protein corona creates a new ‘biological identity’ that can alter their destination in the body [31]. As seen in Figure 3d, there was no change in protein adsorption with increasing F:A molar ratio. With an increase in H:A molar ratio, however, there was a slight reduction in BSA protein adsorption, possibly due to electrostatic repulsion between the nanoparticles and negatively charged BSA protein [47]. Overall, ligand conjugation had negligible impact on protein adsorption which could help limit non-specific targeting of non-target cells. 

### 2.2. In Vitro Cellular Studies

#### 2.2.1. Receptor Expression in Cells

**Figure 4:**
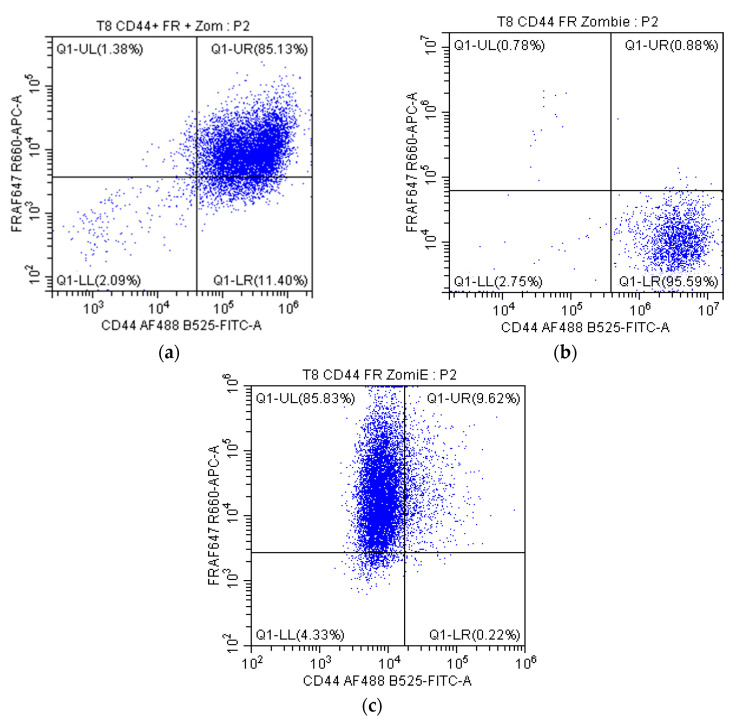
Dot plots for receptor positivity where the x-axis quantifies CD44 and y-axis shows folate receptors. Cells selected for targeting studies included (**a**) SW480 cells, which are positive for both receptors, (**b**) WI38 cells, which were positive for CD44 receptor only, and (**c**) Caco2 cells, which were positive for only the folate receptor.

Prior to conducting cell targeting studies, the receptor expression profiles for cells were determined using flow cytometry. In total, we characterized four colorectal cancer cell lines and three normal cell lines, from which SW470, WI38, and Caco2 were selected because of their varying receptor expression profiles, as shown in Figure 4. Each individual data point in the plots represents a single cell with its location within the quadrants indicating relative CD44 and FR expression. Cells in the top-right quadrant are positive for both receptors, bottom-right are positive only for CD44, bottom-left are negative for both receptors, and top-left are positive only for FR. These three cell lines were selected because SW480 was positive for both receptors, WI38 was positive for only the CD44 receptors, and Caco2 cells were positive for only folate receptors. While it would have been ideal to have a group that did not express these receptors, none of the cell lines evaluated were negative for both CD44 and FR.

#### 2.2.2. Evaluation of Cellular Targeting

**Figure 5:**
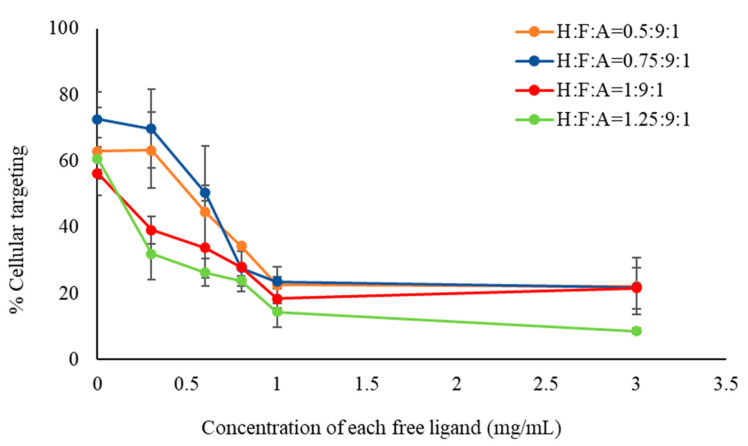
Competitive nanoparticle (0.3 mg/mL) targeting of SW480 cells as a function of free ligand concentration. Pre-incubation cells with free ligand mixture for 3 h resulted in reduced targeting of nanoparticles to cells as the free ligand concentration was increased.

Cellular targeting studies were conducted to evaluate the targeting efficacy of nanoparticles. Initial competitive inhibition studies were conducted by incubating SW480 cells first with free HA and FA (50/50 wt.%) mixture at varying concentrations and then adding 0.3 mg/mL of dual targeted particles for 3 h. As seen in Figure 5, increasing concentrations of the free ligand mixture resulted in increased inhibition of nanoparticle targeting of the cells. Free HA and FA likely bound to the CD44 and FR, respectively, and competed with particle–receptor interactions [33,41,48,49]. Decreased targeting was observed up to a concentration of 1 mg/mL at which it leveled off. These results confirmed that nanoparticle–cellular interactions were based on HA–CD44 receptor and FA–FR interactions.

**Figure 6:**
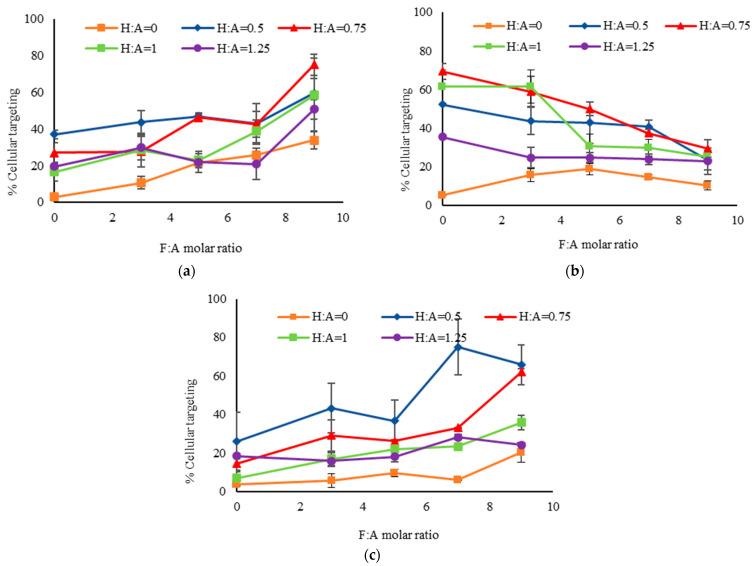
Targeting of silica nanoparticle to (**a**) SW480 cells; (**b**) WI38 cells; and (**c**) Caco2 cells. FR positive cell lines (SW480 and Caco2) showed increased cellular targeting with an increase in F:A molar ratio due to FA-FR mediated endocytosis. CD44 positive cell line WI38 showed reduced cellular targeting with an increase in F:A molar ratio. All 3 cell lines showed increased cellular targeting with an increase in H:A molar ratio up to a certain point and then decreased with further increase in H:A molar ratio up to 1.25 which probably could be due to steric crowding.

Figure 6a shows nanoparticle targeting of SW480 cells, which are positive for both receptors. Each data point represents a different particle formulation with various lines representing the H:A molar ratio, and the F:A ratio is shown on the x-axis. An increase in F:A molar ratio from 0 to 9 produced a significant increase in cellular targeting of nanoparticles. This increase is likely due to FA-FR mediated interactions because SW480 cells are positive for FR [44,50]. With regards to the H:A molar ratio, an optimum was observed at 0.5 for most F:A ratios. Further increase in ligand density yielded lower cellular targeting, likely due to steric crowding of surface ligands restricting interactions with target receptors [15,51,52,53]. 

Nanoparticle targeting of WI38 cells, which are positive only for CD44, is shown in Figure 6b. Each line again represents particles with a specific H:A molar ratio and the x-axis shows the F:A molar ratio. Since WI38 cells do not express the folate receptor, an increase in F:A molar ratio produced particles with reduced cellular targeting. Cellular targeting increased with increasing H:A molar ratio up to a maximum at a ratio of 0.75 beyond which it again decreased. Improved targeting with increasing ligand density is likely due to HA-CD44 mediated endocytosis while its decrease at higher ligand density could be due to steric crowding [15,51,52,53]. 

In Figure 6c, we see the cellular targeting results with Caco2 cells, which are positive only for FR. Increasing the F:A molar ratio produced increased cellular targeting of nanoparticles. There also appeared to be some increase with H:A molar ratio from 0 to 0.5 but it decreased at higher ratios. While the Caco2 cells were highly positive for FR, approximately 10% of the cells showed positivity for CD44, as observed by the few scattered points in the top-right quadrant of Figure 4c. This minor cell population, being positive for FR and CD44, would have exhibited some HA-CD44 mediated targeting by HA-modified particles [54,55]. 

**Figure 7:**
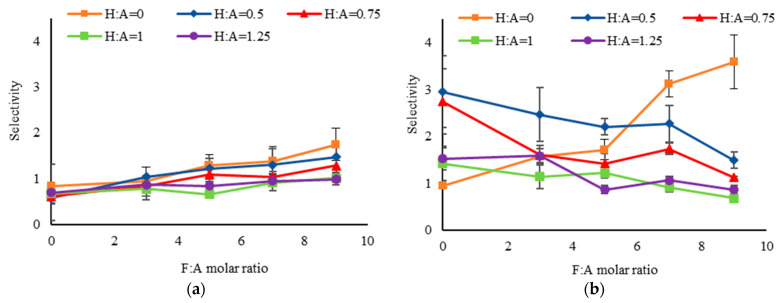
Selectivity of targeted silica nanoparticles comparing (**a**) SW480:WI38; and (**b**) SW480:Caco2. The targeted nanoparticles showed lower selectivity with an increase in H:A molar ratio because both the cancer (SW480) and control cells (WI38 and Caco2) express CD44.

Selectivity of each nanoparticle formulation was calculated as the ratio of colon cancer cell (SW480) targeting relative to targeting of other cells tested. Figure 7a shows the selectivity to SW480 cells, which express both receptors, to WI38 cells that express CD44 only. A higher selectivity was observed with increasing F:A molar ratio, likely due to improved interactions with the cancer cells that express FR. As expected, the selectivity was reduced with increasing H:A molar ratio due to the presence of CD44 on both cell types. Figure 7b compares the selectivity to Caco2 cells which primarily express only FR. Nanoparticles with only FA, marked by the orange line, showed higher selectivity with increasing F:A molar ratio. From Figure 6c, it is observed that with only FA (i.e., H:A = 0) the targeting of Caco2 is uniformly low except for at the highest ratio while the targeting of SW480 (Figure 6a) increases—leading to this observed trend. When HA was added in the reaction mixture, however, the selectivity was reduced with increasing H:A and F:A molar ratios. This opposite trend in selectivity could be due to differences in relative affinity of the two cell types for the targeted nanoparticles. Additionally, the decrease in selectivity with increasing H:A ratio could be a result of the presence of CD44 in approximately 10% of the cells.

## 3. Discussion

The goal of this project was to study the use of dual ligands for targeting of nanoparticles to cells with varying levels of biomarker expression. To achieve this goal, silica nanoparticles were conjugated with hyaluronic acid (HA) and folic acid (FA) to target CD44 and folate receptors, respectively, in a colon cancer model. Following surface modification, the nanoparticles showed negligible protein adsorption and excellent fluorescence stability to allow for in vitro studies with cell cultures. We hypothesized that dual targeting of the CD44 and folate receptors on colon cancer cells would increase the targeting of nanoparticles, compared to single-targeted particles, and that it would be a function of surface ligand density. We observed that cellular targeting was in fact dependent on ligand type, number of ligands used, ligand densities, and cellular expression of target receptors. Cellular targeting was observed to increase as a function of ligand density up to an optimum, beyond which it decreased. In addition, dual targeted nanoparticles showed higher cellular targeting compared to single targeted nanoparticles for cells that positively expressed both receptors. Using cell lines with different expression of the two biomarkers, we have identified different formulations to maximize the targeting and selectivity of nanoparticles for each cell line. Based on FA conjugation, the highest cellular targeting was achieved at F:A = 9 for SW480 and Caco2 cells and at F:A = 0 for WI38 cell. The highest selectivity was achieved at F:A = 9 for both SW480:WI38 and SW480:Caco2 cells. Based on HA conjugation, the highest cellular targeting was achieved at H:A = 0.75 for SW480 and WI38 cells and at H:A = 0.5 for Caco2 cells. The highest selectivity was achieved at H:A = 0 for both SW480:WI38 and SW480:Caco2 cells. Although the single, FR-targeted nanoparticles showed highest selectivity in both cases, the Caco2 cells were shown to also have some expression of the CD44 receptor, which complicated analysis. Additionally, the extent of cellular uptake and any associated toxicity must be taken into account when designing a drug delivery vehicle for therapeutic applications. Moving forward, the targeted nanoparticles developed in this project can be used to encapsulate a therapeutic drug and in vivo and ex vivo experiments used to evaluate the overall anti-tumor efficacy. Successful application of insights gained in this work could lead to improved therapies for colon cancer and other diseases. 

## 4. Materials and Methods

**Materials:** Tetramethyl orthosilicate (TMOS-98%), Triton X-100 and (3-Aminopropyl)trimethoxysilane (APTMS) were purchased from Acros Organics; n-hexanol (99%), fluorescamine, ethanolamine, 1-Ethyl-3-(3 dimethylaminopropyl)carbodiimide (EDC), N-Hydroxysuccinimide (NHS) and fluorescein isothiocyanate were purchased from Alfa Aesar; cyclohexane, aqueous ammonia solution (29 wt.% ammonia) and dimethyl sulfoxide (DMSO) were purchased from BDH Chemicals; ethanol was purchased from Decon Labs; hyaluronic acid (8 kDa) was purchased from DNA Code Skin Care; folic acid (≥97%) was purchased from Enzo Life Sciences; cetrimonium bromide was purchased from Spectrum Chemicals; thiazoyl blue tetrazolium bromide (MTT), sodium acetate was purchased from AMRESCO Inc.; acetic acid was purchased from Macron Fine Chemicals; Coomassie brilliant blue dye was purchased from Bio-Rad; bovine serum albumin was purchased from Sigma Aldrich. 1X Phosphate Buffer Saline was purchased from VWR. DAPI (4′,6-diamidino-2-phenylindole) was purchased from ThermoFisher Scientific. All chemicals were used without further purification. Deionized water used throughout the experiments was purified with an ELGA PURELAB Flex water purification system.

Human Colorectal Adenocarcinoma cells (HT-29, HCT-116, Caco-2 and SW-480), Human Epithelial Prostate (RWPE-1) cells, Human Lung Fibroblast (WI38) cells, and Chinese Hamster Ovary (CHO) cells were purchased from American Type Culture Collection (ATCC). Ham’s F-12K nutrient mixture, 1% penicillin/streptomycin, and 1% L-glutamine were purchased from Corning cellgro; Dulbecco’s Modified Eagles Medium (DMEM) and Eagle’s Minimum Essential Medium (EMEM) were purchased from HyClone; Keratinocyte Serum Free Medium was purchased from Gibco; Fetal Bovine Serum (FBS) was purchased from VWR. Alexa Fluor 488 conjugated Rat (IgG2b,k) monoclonal antibody (Clone IM7, Cat#103015), isotype controls (Clone RTK4530, Cat#400625), zombie violet dye, fixation buffer were purchased from BioLegend. Alexa Fluor 647 conjugated Mouse (IgG1) monoclonal antibody (Clone#548908, Cat#FAB5646R) and its isotype control (Clone#11711, Cat#IC002R) were purchased from R&D Systems (Minneapolis, MN, USA). 

**Nanoparticle synthesis:** Silica nanoparticles were synthesized using the water-in-oil microemulsion method. Initially, a mixture of cyclohexane, n-hexanol, Triton X-100, and DI water was stirred vigorously at room temperature to form a microemulsion. After 15 min, FITC dye was added followed by the addition of TMOS and APTMS after 5 min. NH_4_OH was added after 30 min of vigorous stirring. The reaction was carried on for 24 h at room temperature. Ethanol was added to break the stability of the microemulsion and particles recovered by centrifugation (14,800 rpm for 30 min). Particles were washed three times with ethanol and one time with water to remove any unreacted reagents. Bare silica nanoparticles were stored in DI water at 4 °C prior to use.

Dual-targeted silica nanoparticles with varying densities of the HA and FA ligands on the nanoparticle surface were produced using the well-established EDC-NHS chemistry. In one round bottom flask, hyaluronic acid was activated by dissolving it in deionized water followed by addition of EDC and NHS solutions prepared separately in deionized water. The reaction mixture was stirred overnight. In another round bottom flask, folic acid was activated by dissolving it in DMSO followed by addition of EDC and NHS solutions prepared separately in DMSO. The reaction mixture was stirred overnight in dark. The activated solutions of hyaluronic acid and folic acid were mixed together and stirred. FITC doped amine conjugated silica nanoparticles were suspended in deionized water and then added to the mixture of activated solutions of hyaluronic acid and folic acid and the reaction mixture stirred for 24 h in dark. Following functionalization, the particles were washed 4x with ethanol to remove the unreacted hyaluronic acid and folic acid. To the best of our knowledge, this project presents the first simultaneous approach of HA and FA conjugation on nanoparticle surface. Particles were immediately used for studies.

**Size and Zeta Potential:** Nanoparticle size and zeta potential were measured by dynamic light scattering (DLS) using a Malvern Zetasizer Nano ZS (Malvern, UK). The size was measured with backscatter detection at θ = 173° and zeta potential measured using the Smoluchowski model. 

**Quantification of Amines:** The primary amine content of silica nanoparticles was measured using a quantitative fluorescamine assay. Fluorescamine (4′-phenylspiro [2-benzofuran-3,2′-furan]-1,3′-dione) reacts with primary amines to form fluorescent pyrrolinone moieties. Briefly, 150 µL of nanoparticle suspension was placed into a 96-well plate and 50 µL of 3 mg/mL fluorescamine, dissolved in DMSO, was added to each well and allowed to react for 10 min in dark. Fluorescence of each sample was measured at 400 nm excitation and 460 nm emission with a FlexStation 3 microplate reader (Molecular Devices, Sunnyvale, CA, USA). Ethanolamine of known concentrations was used as a standard.

**Quantification of Hyaluronic Acid:** The hyaluronic acid content of the silica nanoparticles was quantified indirectly using a hexadecyltrimethylammonium bromide (CTAB) turbidimetric method [16]. CTAB is an anionic surfactant that forms an insoluble complex with polyanionic hyaluronic acid. Formation of this complex leads to increased light absorption at 570 nm in a manner that is correlated to the hyaluronic acid concentration. Briefly, 50 μL of supernatant samples after each centrifugation was added in triplicate to a 96-well plate. The samples were incubated with 50 μL of 0.2 M sodium acetate buffer (pH 5.5) at 37 °C for 10 min and then 100 μL of 10 mM CTAB solution was added to the wells. The absorbance of the precipitated complex was read within 10 min against the control using a FlexStation 3 microplate reader (Molecular Devices, Sunnyvale, CA, USA). The amount of conjugated hyaluronic acid was measured by subtracting the total amount of hyaluronic acid in the supernatant solutions from the initial amount added to the reaction mixture. Hyaluronic acid of known concentrations was used as a standard.

**Quantification of Folic Acid:** Folic acid content on the silica nanoparticles was measured using a quantitative ultraviolet (UV) spectrophotometric method at 358 nm. Absorbance of 0.2 mg/mL nanoparticle samples was measured using a FlexStation 3 microplate reader (Molecular Devices, Sunnyvale, CA, USA). Folic acid of known concentrations was used as a standard.

**Determination of Protein Adsorption:** Adsorption of protein on nanoparticles was determined using the Bradford assay with Coomassie brilliant blue dye. Briefly, 0.1 mg/mL of each nanoparticle sample was incubated with 0.5 mg/mL of Bovine Serum Albumin (BSA) at 37 °C and pH 7.4. After 2 h, the mixture was centrifuged at 12,000 rpm for 20 min and 5 µl of supernatant was added to a 96-well plate with 250 µL of the Bradford Reagent. After keeping the mixture at room temperature for 10 min, absorbance of each sample was measured at 595 nm using a FlexStation 3 microplate reader (Molecular Devices, Sunnyvale, CA, USA). The adsorbed BSA was calculated using the equation: *q* = *V*(*C*_i_ − *C*_f_)/*m* where *C*_i_ and *C*_f_ are the initial and final BSA concentrations in the solution, respectively; *V* is the BSA solution volume; and *m* is the mass of nanoparticles added into the solution. BSA of known concentrations was used as a standard.

**Stability of Particle Fluorescence:** The fluorescence stability of nanoparticles was determined by incubating three different concentrations nanoparticles in cell culture media at 37 °C and pH 7.4. At various time points, the fluorescence of each sample was measured at Ex:495nm and Em:525nm using a FlexStation 3 microplate reader (Molecular Devices, Sunnyvale, CA, USA). Culture media without nanoparticle was used as a control. Particle fluorescence was determined to be stable for at least 24 h.

**Visualization by Electron Microscopy:** A Zeiss EM 10 transmission electron microscope (TEM) operating at a voltage of 60 K was used to characterize the shape of the nanoparticles. Samples were prepared by dropping 10 µL of nanoparticle suspension on formvar-carbon film of a 300 mesh copper grid and wiping the remaining solution using a filter paper after 15 min. The grid was then placed in a petri dish and allowed to dry overnight at room temperature.

**Quantification of Receptor Expression:** Expression of CD44 and folate receptor was determined with a BD Accuri C6 flow cytometer (BD Biosciences, San Jose, CA, USA) containing two lasers (488 and 635 nm). The instrument was equipped with a 533/30 band pass filter to examine fluorescence emitted by 488 nm laser excitation and a 675/25 band pass filter to examine florescence emitted by 635 nm laser excitation. Measurements consisted of 10,000 events with a flow rate of 12 μL/min and data recorded for 2 min.

Expression of receptors on cells was determined by first seeding 10^6^ cells/tube, adding 1 μL of zombie violet, and then incubating for 15 min at room temperature in dark. Cells were washed twice with stain buffer and then 5 μL/tube of block buffer was added and incubated for 1 h at room temperature. CD44 or FR antibodies were then added at 100 μg/tube and incubated for 2 h at room temperature in dark. Cells were washed twice with stain buffer before addition of 0.5 mL/tube of fixation buffer and incubation for 1 h at room temperature in dark. Cells were washed twice with stain buffer, re-suspended in 0.5 mL/tube stain buffer, and then filtered through a 40 μm filter. Cells were kept in the dark at 4 °C before analysis by flow cytometry. Unstained cells and cells with isotype antibodies were used as controls.

**Maintenance of Cell Cultures:** Chinese Hamster Ovary (CHO) cells were maintained in Ham’s F-12K nutrient mixture with L-glutamine, supplemented with 10% fetal bovine serum and 1% penicillin. Human Colon Adenocarcinoma (HCT116) cells were maintained in Dulbecco’s Modified Eagles Medium, supplemented with 10% FBS and 1% antibiotics. Human Colorectal Adenocarcinoma cells (HT-29, HCT-116 and SW-480) were maintained in Dulbecco’s Modified Eagle’s Medium, supplemented with 10% FBS, 1% L-glutamine, and 1% antibiotics. Human Epithelial Prostate (RWPE-1) cells were maintained in Keratinocyte Serum Free Medium supplemented with EGF and BPE. Human Fibroblast (WI38) cells were maintained in Eagle’s Minimum Essential Medium, supplemented with 10% FBS and 1% antibiotics. Human Colorectal Adenocarcinoma (Caco-2) cells were maintained in Eagle’s Minimum Essential Medium, supplemented with 20% FBS and 1% antibiotics. Human Embryonic Kidney (HEK-293) cells were maintained in Eagle’s Minimum Essential Medium, supplemented with 10% FBS. All cell lines were incubated at 37 °C in 5% CO_2_.

**Assessment of Cytotoxicity:** Chinese Hamster Ovary (CHO) cells were seeded in a 96-well plate at 20,000 cells/well and incubated overnight at 37 °C. After incubation, the culture media was renewed with culture media containing varying concentrations of nanoparticles. Four hours prior to each measurement time point, 2 mg/mL of MTT was added to each well and, after 4 h, the culture media of each well aspirated completely without touching the blue-purple crystals of insoluble formazan. DMSO was added to each well to dissolve the crystals and the well plate was vortexed for 5 min, at around 500 rpm, with the plate agitator to completely dissolve the crystals. Absorbance was measured at 540 nm using a spectrophotometer. Control samples consisted of cells without nanoparticle treatment. Cell viability was calculated as the ratio of the absorbance of cells with nanoparticles divided by absorbance of control cells) × 100%. Effects of particles on cells were negligible with all samples showing viability ≥ 90% at concentrations up to 100 µg/mL (data not shown).

**Cellular Targeting Studies:** For qualitative analysis of cellular targeting, cells were seeded at 100,000 cells/well in 6-well plates and incubated overnight at 37 °C. The culture media was replaced with culture media containing varying concentrations of nanoparticles and incubated for a desired time period. Cells were then rinsed thrice with ice-cold 1X PBS (pH 7.4, 4 °C) to eliminate excess nanoparticles and dead cells. Then, the cells were fixed with 4% paraformaldehyde for 1 h at room temperature and rinsed thrice with ice-cold 1X PBS (pH 7.4, 4 °C). The cells were stained with 300 nM DAPI solution for 5 min in the dark at room temperature and again rinsed thrice with ice-cold 1X PBS (pH 7.4, 4 °C). Finally, the cells were imaged under a fluorescence microscope. Cells without nanoparticle treatment were used as control.

For quantitative analysis of cellular targeting, 40,000 cells/well were seeded in a 96-well plate and incubated overnight at 37 °C. The culture media was then replaced with culture media containing varying concentrations of nanoparticles, which were incubated for a predetermined amount of time. The media was then aspirated and transferred to a different 96 well-plate for analysis. The cells were thoroughly rinsed thrice with ice-cold 1X PBS (pH 7.4, 4 °C) to eliminate excess nanoparticles and dead cells and the cells lysed using lysis buffer for 60 min at room temperature. After lysis, the well plate was vortexed for 5 min at 500 rpm and the fluorescence of FITC associated with the cells and the previously collected media measured at excitation of 495 nm and emission of 525 nm. The fluorescence intensity was converted to number of nanoparticles based on a standard curve obtained with known nanoparticle concentrations in the lysis buffer and culture media. Cells without nanoparticle treatment were used as control.

Cellular targeting of each nanoparticle sample was calculated using the equation,

$$\%\;\mathrm{Targeted}=\frac{Fluorescence\;of\;cell\;associated\;nanoparticle}{Fluorescence\;of\;nanoparticle\;added}\times 100\%$$

Selectivity of each nanoparticle sample was calculated using the equation,

$$\mathrm{Selectivity}=\frac{Concentration\;of\;nanoparticle\;targeted\;to\;SW480\;cell}{Concentration\;of\;nanoparticle\;targeted\;to\;WI38\;or\;Caco2\;cell}$$

Distribution co-efficient of each nanoparticle sample was calculated using the equation,

$$\mathrm{Distribution}\;\mathrm{co}\text{-}\mathrm{efficient}=\frac{Concentration\;of\;nanoparticle\;targeted\;to\;cell}{Concentration\;of\;nanoparticle\;in\;the\;media}$$

The same protocol was followed for the competitive inhibition study with the exception that cells were preincubated with different concentrations of free ligand mixture for 3 h before nanoparticle addition.

## Figures and Tables

**Table 1:** Summary of synthesis conditions and resulting size and polydispersity index (PDI) of targeted nanoparticles.

Molar Ratios		Hydrodynamic Diameter (nm)	PDI
H:A	F:A		
0	0	271 ± 4	0.23 ± 0.02
	3	264 ± 3	0.21 ± 0.01
	5	261 ± 3	0.23 ± 0.01
	7	247 ± 2	0.21 ± 0.01
	9	234 ± 2	0.20 ± 0.01
0.5	0	269 ± 5	0.15 ± 0.02
	3	156 ± 3	0.07 ± 0.04
	5	155 ± 2	0.12 ± 0.02
	7	207 ± 2	0.22 ± 0.01
	9	225 ± 1	0.22 ± 0.02
0.75	0	155 ± 4	0.16 ± 0.01
	3	219 ± 7	0.20 ± 0.01
	5	194 ± 3	0.16 ± 0.01
	7	193 ± 3	0.19 ± 0.02
	9	252 ± 6	0.22 ± 0.01
1	0	198 ± 5	0.13 ± 0.01
	3	147 ± 2	0.28 ± 0.02
	5	239 ± 1	0.24 ± 0.01
	7	195 ± 1	0.17 ± 0.04
	9	250 ± 2	0.23 ± 0.03
1.25	0	211 ± 4	0.12 ± 0.02
	3	231 ± 3	0.24 ± 0.02
	5	164 ± 1	0.21 ± 0.02
	7	181 ± 2	0.16 ± 0.03
	9	193 ±2	0.19 ± 0.01

## Data Availability

Not applicable.

## References

[1] Shi M., Ho K., Keating A., Shoichet M.S. (2009). Doxorubicin-Conjugated Immuno-Nanoparticles for Intracellular Anticancer Drug Delivery. Adv. Funct. Mater..

[2] Banerjee A., Pathak S., Subramanium V.D., Dharanivasan G., Murugesan R., Verma R.S. (2017). Strategies for targeted drug delivery in treatment of colon cancer: Current trends and future perspectives. Drug Discov. Today.

[3] Danhier F., Feron O., Préat V. (2010). To exploit the tumor microenvironment: Passive and active tumor targeting of nanocarriers for anti-cancer drug delivery. J. Control. Release.

[4] Bazak R., Houri M., El Achy S., Kamel S., Refaat T. (2015). Cancer active targeting by nanoparticles: A comprehensive review of literature. J. Cancer Res. Clin. Oncol..

[5] Cisterna B.A., Kamaly N., Choi W.I., Tavakkoli A., Farokhzad O.C., Vilos C. (2016). Targeted nanoparticles for colorectal cancer. Nanomedicine.

[6] Jokerst J.V., Gambhir S.S. (2011). Molecular Imaging with theranostic nanoparticles. Acc. Chem. Res..

[7] Peiris P., He F., Covarrubias G., Raghunathan S., Turan O., Lorkowski M., Gnanasambandam B., Wu C., Schiemann W.P., Karathanasis E. (2018). Precise targeting of cancer metastasis using multi-ligand nanoparticles incorporating four different ligands. Nanoscale.

[8] Zhang Y., Li Y., Tian H., Zhu Q., Wang F., Fan Z., Zhou S., Wang X., Xie L., Hou Z. (2019). Redox-Responsive and Dual-Targeting Hyaluronic Acid− Methotrexate Prodrug Self-Assembling Nanoparticles for Enhancing Intracellular Drug Self-Delivery. Mol. Pharm..

[9] Liu Y., Sun J., Cao W., Yang J., Lian H., Li X., Sun Y., Wang Y., Wang S., He Z. (2011). Dual targeting folate-conjugated hyaluronic acid polymeric micelles for paclitaxel delivery. Int. J. Pharm..

[10] Carlson C.B., Mowery P., Owen R.M., Dykhuizen E.C., Kiessling L.L. (2007). Selective Tumor Cell Targeting Using Low-Affinity, Multivalent Interactions. ACS Chem. Biol..

[11] Montet X., Funovics M., Montet-Abou K., Weissleder R., Josephson L. (2006). Multivalent Effects of RGD Peptides Obtained by Nanoparticle Display. J. Med. Chem..

[12] Gantert M., Lewrick F., Adrian J.E., Rössler J., Steenpass T., Schubert R., Peschka-Süss R. (2009). Receptor-Specific Targeting with Liposomes In Vitro Based on Sterol-PEG 1300 Anchors. Pharm. Res..

[13] Lu B., Smytha M.R., O’Kennedy R. (1996). Oriented Immobilization of Antibodies and Its Applications in Immunoassays and Immunosensors. Analyst.

[14] Jahan S.T., Sadat S.M.A., Walliser M., Haddadi A. (2017). Targeted Therapeutic Nanoparticles: An Immense Promise to Fight against Cancer. J. Drug Deliv..

[15] Anzengruber M., Nepustil L., Kurtaj F., Tahir A., Skoll K., Sami H., Wirth M., Gabor F. (2023). A Versatile Brij-Linker for One-Step Preparation of Targeted Nanoparticles. Pharmaceutics.

[16] Syed H., Qhattal S., Liu X. (2011). Characterization of CD44-Mediated Cancer Cell Uptake and Intracellular Distribution of Hyaluronan-Grafted Liposomes. Mol. Pharm..

[17] Moradi E., Vllasaliu D., Garnett M., Falcone F., Stolnik S. (2012). Ligand density and clustering effects on endocytosis of folate modified nanoparticles. RSC Adv..

[18] Emami F., Duwa R., Banstola A., Woo S.M., Kwon T.K. (2023). Dual receptor specific nanoparticles targeting EGFR and PD-L1 for enhanced delivery of docetaxel in cancer therapy. Biomed. Pharmacother..

[19] Liberman A., Mendez N., Trogler W.C., Kummel A.C. (2014). Synthesis and surface functionalization of silica nanoparticles for nanomedicine. Surf. Sci. Rep..

[20] Jung H.-S., Moon D.-S., Lee J.-K. Quantitative Analysis and Efficient Surface Modification of Silica Nanoparticles. J. Nanomater..

[21] He X., Nie H., Wang K., Tan W., Wu X., Zhang P. (2003). In Vivo Study of Biodistribution and Urinary Excretion of Surface-Modified Silica Nanoparticles. Proc. Natl. Acad. Sci. USA.

[22] Watermann A., Brieger J. (2017). Mesoporous Silica Nanoparticles as Drug Delivery Vehicles in Cancer. Nanomaterials.

[23] Zhou Y., Quan G., Wu Q., Zhang X., Niu B., Wu B., Huang Y., Pan X., Wu C. (2018). Mesoporous silica nanoparticles for drug and gene delivery. Acta Pharm. Sin. B.

[24] Yu M.K., Park J., Sangyong J. (2012). Targeting Strategies for Multifunctional Nanoparticles in Cancer Imaging and Therapy. Theranostics.

[25] Ud Din F., Aman W., Ullah I., Qureshi O.S., Mustapha O., Shafique S., Zeb A. (2017). Effective use of nanocarriers as drug delivery systems for the treatment of selected tumors. Int. J. Nanomed..

[26] Toporkiewicz M., Meissner J., Matusewicz L., Czogalla A., Sikorski A.F. (2015). Toward a magic or imaginary bullet? Ligands for drug targeting to cancer cells: Principles, hopes, and challenges. Int. J. Nanomed..

[27] Hobbs S.K., Monsky W.L., Yuan F., Roberts W.G., Griffith L., Torchilin V.P., Jain R.K. (1998). Regulation of transport pathways in tumor vessels: Role of tumor type and microenvironment. Proc. Natl. Acad. Sci. USA.

[28] Bae Y.H. (2009). Drug targeting and tumor heterogeneity. J. Control. Release.

[29] Kirpotin D.B., Drummond D.C., Shao Y., Shalaby M.R., Hong K., Nielsen U.B., Marks J.D., Benz C.C., Park J.W. (2006). Antibody Targeting of Long-Circulating Lipidic Nanoparticles Does Not Increase Tumor Localization but Does Increase Internalization in Animal Models. Cancer Res..

[30] Pirollo K.F., Chang E.H. (2008). Does a targeting ligand influence nanoparticle tumor localization or uptake?. Trends Biotechnol..

[31] Alkilany A.M., Farokhzad O.C., Mahmoudi M., Behzadi S., Serpooshan V., Tao W., Hamaly M.A., Alkawareek M.Y., Dreaden E.C., Brown D. (2017). Cellular uptake of nanoparticles: Journey inside the cell. Chem. Soc. Rev..

[32] Shi Y., Massagué J. (2003). Mechanisms of TGF-β signaling from cell membrane to the nucleus. Cell.

[33] Wang T., Hou J., Su C., Zhao L., Shi Y. (2017). Hyaluronic acid-coated chitosan nanoparticles induce ROS-mediated tumor cell apoptosis and enhance antitumor efficiency by targeted drug delivery via CD44. J. Nanobiotechnology.

[34] Patel L.N., Zaro J.L., Shen W.-C. (2007). Expert Review Cell Penetrating Peptides: Intracellular Pathways and Pharmaceutical Perspectives. Pharm. Res..

[35] Conner S.D., Schmid S.L. (2003). Regulated portals of entry into the cell. Nature.

[36] Kumari S., Mg S., Mayor S. (2010). Endocytosis unplugged: Multiple ways to enter the cell. Cell Res..

[37] Contini C., Schneemilch M., Gaisford S., Quirke N. (2018). Nanoparticle-membrane interactions. J. Exp. Nanosci..

[38] Rojas-Chapana J.A., Correa-Duarte M.A., Ren Z., Kempa K., Giersig M. (2023). Enhanced Introduction of Gold Nanoparticles into Vital *Acidothiobacillus ferrooxidans* by Carbon Nanotube-based Microwave Electroporation. Nano Lett..

[39] Hillaireau H., Couvreur P. (2009). Nanocarriers’ entry into the cell: Relevance to drug delivery. Cell. Mol. Life Sci..

[40] Jiang X., Rö C., Hafner M., Brandholt S., Dö R.M., Nienhaus G.U. (2010). Endo- and Exocytosis of Zwitterionic Quantum Dot Nanoparticles by Live HeLa Cells. ACS Nano.

[41] Tabata Y., Ikada Y. (1988). Effect of the size and surface charge of polymer microspheres on their phagocytosis by macrophage. Biomaterials.

[42] Gustafson H.H., Holt-Casper D., Grainger D.W., Ghandehari H. (2015). Nanoparticle uptake: The phagocyte problem. Nano Today.

[43] Aubert T., Grasset F., Mornet S., Duguet E., Cador O., Cordier S., Molard Y., Demange V., Mortier M., Haneda H. (2010). Functional silica nanoparticles synthesized by water-in-oil microemulsion processes. J. Colloid Interface Sci..

[44] Zhou Z., Zhang C., Qian Q., Ma J., Huang P., Zhang X., Pan L., Gao G., Fu H., Fu S. (2013). Folic acid-conjugated silica capped gold nanoclusters for targeted fluorescence/X-ray computed tomography imaging. J. Nanobiotechnol..

[45] Alvarez-Berríos M.P., Vivero-Escoto J.L. (2016). In vitro evaluation of folic acid-conjugated redox-responsive mesoporous silica nanoparticles for the delivery of cisplatin. Int. J. Nanomed..

[46] Zhang W., Wang Y., Li Z., Wang W., Sun H., Liu M. (2018). Synthesis and Characterization of Hyaluronic Acid Modified Colloidal Mesoporous Silica Nanoparticles. IOP Conf. Ser. Mater. Sci. Eng..

[47] Zhao Q., Geng H., Wang Y., Gao Y., Huang J., Wang Y., Zhang J., Wang S. (2014). Hyaluronic Acid Oligosaccharide Modified Redox-Responsive Mesoporous Silica Nanoparticles for Targeted Drug Delivery. ACS Appl. Mater. Interfaces.

[48] Feng S., Zhang H., Xu S., Zhi C., Nakanishi H., Gao X.D. (2019). Folate-conjugated, mesoporous silica functionalized boron nitride nanospheres for targeted delivery of doxorubicin. Mater. Sci. Eng. C.

[49] Cheng W., Nie J., Xu L., Liang C., Peng Y., Liu G., Wang T., Mei L., Huang L., Zeng X. (2017). pH-Sensitive Delivery Vehicle Based on Folic Acid-Conjugated Polydopamine-Modified Mesoporous Silica Nanoparticles for Targeted Cancer Therapy. ACS Appl. Mater. Interfaces.

[50] Yang H., Lou C., Xu M., Wu C., Miyoshi H., Liu Y. (2011). Investigation of folate-conjugated fluorescent silica nanoparticles for targeting delivery to folate receptor-positive tumors and their internalization mechanism. Int. J. Nanomed..

[51] Cao J., Zhang Y., Wu Y., Wu J., Wang W., Wu Q., Yuan Z. (2018). The effects of ligand valency and density on the targeting ability of multivalent nanoparticles based on negatively charged chitosan nanoparticles. Colloids Surf. B Biointerfaces.

[52] Alkilany A.M., Zhu L., Weller H., Mews A., Parak W.J., Barz M., Feliu N. (2019). Ligand density on nanoparticles: A parameter with critical impact on nanomedicine. Adv. Drug Deliv. Rev..

[53] Elias D.R., Poloukhtine A., Popik V., Tsourkas A. (2013). Effect of ligand density, receptor density, and nanoparticle size on cell targeting. Nanomedicine.

[54] Saneja A., Nayak D., Srinivas M., Kumar A., Khare V., Katoch A., Goswami A., Vishwakarma R.A., Sawant S.D., Gupta P.N. (2017). Development and mechanistic insight into enhanced cytotoxic potential of hyaluronic acid conjugated nanoparticles in CD44 overexpressing cancer cells. Eur. J. Pharm. Sci..

[55] Edelman R., Assaraf Y.G., Levitzky I., Shahar T., Livney Y.D. (2017). Hyaluronic acid-serum albumin conjugate-based nanoparticles for targeted cancer therapy. Oncotarget.

